# The College of Nursing

**Published:** 1916-06-24

**Authors:** 


### THE COLLEGE OF NURSING.

### MEETING OF REPRESENTATIVES OF NURSE-TRAINING SCHOOLS.

### Full Report.

A meeting to promote the formation of a Consultative Board of the College of Nursing was held at St. Thomas's Hospital on Thursday, June 15, 1916, the Hon. Arthur Stanley, M.P., C.B., M.V.O., in the Chair.

### THE AUDIENCE.

The Hon. Arthur Stanley, M.P., C.B., M.V.O. (Chairman), Sir Cooper Perry, M.D., Dr. Horace Turney, Miss S. A. Swift, R.R.C., Mrs. L. V. Harcourt, Mabell Countess of Airlie, Dr. Sidney Phillips, Miss Amy Hughes, the Hon. Hermione Blackwood, Lady Miller, Miss Musson, R.R.C., Miss E. Mowat, Dr. Everitt E. Norton, Miss Esther Young (Windsor), and the following:

Metropolitan Training Schools and Hospitals.

*Alexandra Hospital, Queen Square*: Miss E. Fitch (matron). *Brompton Hospital for Consumption*: Miss F. T. Rede (matron). *Bolingbroke Hospital*: Miss M. Foster (matron). *City of London Hospital for Diseases of the Chest*: Dr. Arnold Chaplin, Miss L. G. Dalton (matron). *Evelina Hospital for Children*: Miss F. Goode (sister-matron). *Great Northern Central Hospital*: Miss Bird. *Guy's Hospital*: Mr. Cosmo Bonsor (treasurer), Miss L. V. Haughton (matron). *Hospital for Sick Children, Great Ormond Street, W.C.*: Dr. A. F. Voelcker, Miss G. Payne (matron), Mr. Jas. McKay (acting secretary). *King's College Hospital*: Col. C. F. Fellows. *London Homœopathic Hospital*: Mr. Edwin A. Neatby, Miss M. E. Belsham (matron). *Middlesex Hospital*: Mr. John Murray, F.R.C.S., Miss M. G. Montgomery (matron), *Nightingale Fund*: Mr. W. H. Bonham Carter (secretary). *Prince of Wales' General Hospital*: Viscount Gladstone, Dr. R. Murray Leslie. *Royal Free Hospital*: Dr. G. Hudleston, Mr. Herbert M. Phipson. *St. Bartholomew's Hospital*: Miss A. McIntosh (matron). *St. George's Hospital*: Miss E. Cooper (matron). *St. Mary's Hospital*: Miss R. E. Darbyshire, R.R.C. (matron). *University College Hospital*: Captain T. D. Butler, Miss D. Finch (matron). *West Ham and Eastern General Hospital*: Miss E. A. Sordy (matron). *West London Hospital*: Mr. Aslett Baldwin, F.R.C.S., Miss F. E. Nevile (matron).

Metropolitan Poor-Law Infirmaries and Unions.

*City of Westminster Union*: Miss Margaret Torrey. *City of Westminster Infirmary*: Miss A. E. Booth (matron). *Edmonton Union*: Mrs. Cole. *Fulham Infirmary*: Dr. C. Thackray Parsons. *Hackney Union*: Miss Griffith. *Hammersmith Union*: Mr. W. Elliott (chairman). *Hampstead Union*: Mrs. M. A. Munro (vice-chairman), Mr. E. S. Payne. *Kensington Infirmary*: Miss H. A. Alsop (matron). *Lambeth Union*: Mr. F. Briant, Dr. A. L. Baly (medical officer), Mr. James J. L. Goldspink (clerk). *Mile End Union*: Miss J. H. Lilley. *Paddington Union*: Mrs. Mylne. *Paddington Infirmary*: Miss G. E. Copeman (matron). *St. George's-in-the-East Union*: Miss H. L. Cartwright. *St. Marylebone Infirmary*: Miss S. J. Cockrell (matron), Mrs. J. O. Walshe. *St. Pancras Union*: Mrs. E. E. Lidgett. *St. Pancras North Infirmary*: Mrs. E. Hopkins. *Southwark Infirmary*: Dr. H. W. Bruce (medical superintendent). *Wandsworth Union*: Rev. Canon Curtis (chairman), Dr. Clara A. S. Fitter. *West Ham Infirmary*: Mr. Andrew Stewart, Miss Clark (matron). *Whitechapel Union*: F. Tootell.

Provincial Training Schools, Hospitals, Infirmaries, and Unions.

*Ashton-under-Lyne District Infirmary*: Miss C. F. Cooper (matron). *Ashton-under-Lyne Union Hospital*: Miss C. Seymour Yapp (matron). *Bath Royal United Hospital*: Miss F. Mason (matron). *Bedford County Hospital*: Mr. J. A. Whitchurch (chairman), Miss A. L. Charteris (matron). *Birmingham General Hospital*: Mr. J. Edward Willcox. *Birmingham, Selly Oak Infirmary*: Miss A. M. E. Bodley (matron). *Bolton Union*: Mr. Shippobottom, Mr. Cooper. *Brighton, Royal Sussex County Hospital*: Captain T. D. Butler, Mr. R. Ball Dodson (chairman), Miss K. Scott (matron). *Bristol General Hospital*: Miss A. E. Densham (matron). *Bristol Royal Infirmary*: Mr. J. N. C. Pope, Miss A. B. Baillie, R.R.C. (matron), Mr. W. E. Budgett (secretary). *Burnley, Victoria Hospital*: Mr. F. A. Hargreaves (hon. secretary), Miss J. Lindsay (matron). *Bradford Union*: Mr. R. S. Dawson, Mr. G. M. Crowther (clerk). *Cardiff Union*: Dr. J. D. Williams, Miss C. M. Williams. *Cheltenham General Hospital*: Miss Falconer (matron). *Chester Royal Infirmary*: Miss E. K. Blayney (matron). *Coventry and Warwickshire Hospital*: Dr. T. Webb Turner, Miss S. Hutchinson (matron). *Croydon Infirmary*: Mr. R. W. Wilson (medical superintendent). *Croydon Union*: Mr. H. List. *Derbyshire Royal Infirmary*: Admiral Sir F. Inglefield, Miss Sutcliffe (matron). *Epsom Union*: Mr. A. W. Ebbut (clerk), Mr. R. Homan. *Exeter, Royal Devon and Exeter Hospital*: Mr. Edward J. Domville. *Ipswich, East Suffolk Hospital*: Miss M. Deane (matron). *Leamington, Warneford Hospital*: Mr. Allan E. Batchelor, Miss K. F. M. Jackson (matron). *Leeds General Infirmary*: Mr. Charles Lupton (treasurer). *Leeds, Hunslet Union*: Mr. William A. Appleyard, Mr. F. D. Mee. *Leeds Township Infirmary*: Miss F. Spann (matron). *Leicester Royal Infirmary*: Miss C. E. Vincent, R.R.C. (matron). *Leicester, North Evington Poor-Law Infirmary*: Dr. E. C. Hadley (medical superintendent), Miss Masters (matron). *Lincoln County Hospital*: Miss J. A. Sheppard, R.R.C. (matron). *Liverpool Royal Infirmary*: Mr. H. Wade Deacon (chairman), Miss Margaret Cummins (lady superintendent). *Liverpool Royal Southern Hospital*: Miss Agnes Bagnall (acting matron). *Liverpool Select Vestry*: Mr. T. H. Nabb (chairman), Miss J. Thorburn. *Liverpool, West Derby Union*: Mr. John Ellis, Mr. R. W. Maddox (deputy clerk). *Manchester, Ancoats Hospital*: Mr. H. J. Dafforne (secretary). *Manchester (Crumpsall Union)*: Mr. O. Hertz, Mrs. C. E. M. Garrett. *Manchester, Royal Infirmary*: Miss M. E. Sparshott, R.R.C. (matron). *Newport (Mon.), Royal Gwent Hospital*: Sir Garrod Thomas, M.D. *Newport (Mon.) Union*: Miss M. C. Jones, Mr. G. H. Cook. *Northampton General Hospital*: Miss L. Atkinson (matron). *Norfolk and Norwich Hospital*: Miss Florence A. Cann (matron). *Oxford, Radcliffe Infirmary*: Mrs. T. H. Green.

**THE COLLEGE OF NURSING: Full Report**—(*continued*).

*Portsmouth Royal Hospital*: Miss C. Alcock (matron). *Portsmouth Workhouse Infirmary*: Miss F. A. Foster. *Reading, Royal Berks Hospital*: Dr. Gell, Miss E. A. Wynne (matron). *Rochester, St. Bartholomew's Hospital*: Miss M. Pote-Hunt (matron). *Salford Poor-Law Infirmary*: Mr. J. Dudgeon Giles (medical superintendent), Mr. Jas. Dewhurst. *Sheffield Union*: The Rev. S. E. Day. *Sheffield, Ecclesall Bierlow Union Infirmary*: Mr. James Blossom, Mr. G. J. French. *Shrewsbury, Royal Salop Infirmary*: Miss E. S. Garside (matron). *Stockport Union*: Messrs. Henry Pattern and John Chetham. *Stoke-on-Trent, North Staffs Infirmary*: Major T. Basil Rhodes (secretary and house governor). *Stoke-on-Trent Union*: Rev. Arthur S. Langley, Mrs. F. G. Beardmore. *Sunderland Union Hospital*: Miss H. A. Clark. *Swansea General Hospital*: Miss M. P. Scovell (matron). *Swansea Union*: M. L. Jenkins. *Tynemouth Union*: Mr. Tom Percival (clerk). *Tynemouth Union Infirmary*: Miss A. Flick (matron). *Wakefield, Clayton Hospital*: Miss Dora Pressland (matron). *Warrington Union*: Mr. A. Bottomley (clerk), Mr. Jolley. *West Bromwich Union*: Miss S. Mold, Mr. J. O. Maule Smith. *Wigan Royal Albert Edward Infirmary*: Mr. S. M. Lamb. *Winchester, Royal Hants County Hospital*: Mr. J. C. Warner (chairman), Miss E. C. Carpenter Turner (matron). *Withington Hospitals*: Miss E. M. Smith (lady superintendent). *Wolverhampton General Hospital*: Mr. W. H. Harper (secretary), Miss H. Hannath (matron). *Worcester General Infirmary*: Miss Mary Herbert (matron). *York County Hospital*: Miss K. S. Stewart (matron).

Nursing Associations and Institutions.

*Derby, Nightingale Nursing Home*: M. Scott. *Derby Royal Nursing Association*: Miss Agnes Atthill (lady superintendent). *Highfield Nursing Institute, Edmonton*: Miss E. B. Benjafield (matron). *Jewish Maternity Sick-room Helps Society*: Misses H. F. Walter, R. F. Hertz, M. Gration. *National Union of Trained Nurses*: Misses V. Thurstan, E. M. Cancellor, E. L. C. Eden. *North-Western Poor-Law Conference*: Mr. Charles E. Bygrave (Blackburn Union), Mr. R. H. Leach. *Royal National Pension Fund for Nurses*: Mr. Charles Coburn. *Royal British Nurses' Association*: Dr. W. Bezly Thorne, Miss I. Macdonald (secretary).

Training Schools, Scotland.

*Dundee Royal Infirmary*: Mr. C. C. Barrie. *Edinburgh Royal Infirmary*: Miss A. W. Gill, R.R.C. *Glasgow Western Infirmary*: Mr. Nicol P. Brown, Miss H. Gregory Smith, R.R.C. (matron).

Training Schools, Ireland.

*Belfast Royal Victoria Hospital*: Miss M. F. Bostock.

Military and Territorial Hospitals.

*Mile End Military Hospital*: Mr. A. Alan. *Richmond Military Hospital*: Miss Gertrude Fletcher (matron). *New Zealand Military Hospital*: Miss Florence H. Redding. *Territorial Force Nursing Service*: Miss Sidney Browne, R.R.C. (Matron-in-Chief). *2nd London General Hospital*: Miss M. S. Riddell (matron). *4th London General Hospital*: Miss E. G. Taylor. *2nd Western General Hospital, Manchester*: Miss E. E. Fletcher.

### The Chairman's Speech.

The Hon. Arthur Stanley (who on rising was greeted with applause) said: Ladies and Gentlemen,—They say there is nothing like beginning as you mean to go on, and we will therefore begin by being frankly out of order. (Laughter.) We have got a friend here to-day, Mr. Cosmo Bonsor, who has probably done more for nurses and nursing than anyone else. He has a boy going off to the Front, and is obliged to go early; but before he does go I would like to ask him to give one word of blessing—I hope it is going to be blessing—to our scheme.

Mr. Cosmo Bonsor: Mr. Stanley, it is a very great compliment, I can assure you, which you have paid me, and, ladies and gentlemen, I cannot resist an invitation of that description. It is perfectly true that for five-and-thirty years I have had the honour of being a Governor of Guy's Hospital, and that during that time I have taken a keen interest in what I might say is the welfare of the nursing profession. I have been very disappointed frequently by the fact that the professional nurses of our country, who give their time and such devotion to the sufferings of humanity, should never have received either State recognition or even that recognition which their devotion requires from ourselves. Therefore I am only too pleased to be here on this platform to-day and to see this large meeting, who, I feel sure, are anxious to see that the profession of nursing is at last properly recognised as one of the great professions of this country—recognised by the State, recognised by us all, as being one that should be in our minds as one of the most useful and the most agreeable of the professions in our country. Sir, I am very grateful to you for having allowed me to intervene. It is true that I have just got to go to the train to see a boy off to the Continent; he has been here on a short leave, and I am naturally anxious to be allowed to shake hands with him on his departure. (Applause.)

The Chairman: Ladies and Gentlemen,—I have been obliged to put it on the notice convening the meeting that, in view of the possibly large number of people who may wish to speak, speeches should be limited to five minutes, and although I do not promise to keep within the five minutes myself I shall do my best to set the example, at all events, of being brief. Well, luckily, I do not think that I need go into the whole question of how this College came to be founded. I am glad to say that it is actually in being, and we therefore need not discuss the origin of its being. What we have got to discuss now, and what we want your opinion about, is how it is to be made the most effective instrument for securing the recognition of nurses and the status of the nursing profession.

Let me say in passing that we have received a certain amount of criticism from the fact that we started it as a limited liability company. We did this for a very simple reason. We had to get some kind of body in being in order that we might get to work on the schemes which we have in connection with the College. The simple way to do that was to go to the Board of Trade and get registered as a limited concern. Once we get our Bill, about which I shall speak later, that word "Limited" will drop out, and you will cease to have that stigma—(laughter)—and, as I shall explain later, I hope we shall eventually find ourselves "The Royal College of Nursing" without "Limited."

### The Royal British Nurses' Association and the College.

Now, the objects of the College are all set out in the leaflet which I think you have received. I will not go into those. I think you will all agree with them. They are: "(1) To promote the better education and training of nurses and the advancement of nursing as a profession in all or any of its branches; (2) to promote uniformity of curriculum; (3) to recognise approved training schools; (4) to make and maintain a register of nurses; and (5) to promote Bills in Parliament for any object connected with the interests of nurses, and, in particular, with their education, organisation, protection, and for their recognition by the State." Now, there was an association formed a good many years ago with very much the same objects, and that was the Royal British Nurses' Association, and I have been asked why we did not simply strengthen that body, or, rather, get that body to take up this movement vigorously. Well, my answer is that times have moved since then, and that the objects which we seek to obtain are somewhat broader than those which are mentioned in their Royal Charter. But I am glad to say that Dr. Bezly Thorne, their Chairman, has been kind enough to become one of our Vice-Presidents, and Dr. Comyns Berkeley, their Treasurer, is also our Honorary Treasurer. We have had several conferences, and I have every reason to hope that we shall be able to come to an agreement. Her Royal Highness Princess Christian, as you probably know, is the President of that Association, and she has authorised me to say that "should a satisfactory scheme of union between the College of Nursing and the Royal British Nurses' Association be formulated, H.R.H. the Princess Christian, President of the Royal British Nurses' Association"—and this little bit is what we have added—who has for so long past interested herself in the cause of nurses and the progress of the nursing profession—"would be disposed to accept a position of honour in the conjoint Society." We are therefore assured beforehand of the good will and the assistance of the one institution that has got somewhat the same objects as ours—the Royal British Nurses' Association.

### The Draft Registration Bill.

Well, now, as set out in the letter of invitation to this meeting, we have got three things to discuss to-day. The first is the draft Bill for the Registration of Nurses. I will take that first. As you are aware, there has already been before Parliament a Bill for the registration of nurses. That Bill was promoted by the Central Committee for the State Registration of Trained Nurses. We have had several conferences with the representatives of that Committee, and although I cannot absolutely present this to you as an agreed Bill, I can say that we have gone very far towards agreement, and that substantially this represents the outcome of our conferences. You will see that this College of Nursing, Limited, will be authorised to drop the word "Limited," and will be called the General Nursing Council and College of Nursing.

### Qualifications for Admission to Register.

It is intended at once to proceed to the formation of a Register of Trained Nurses. The conditions under which nurses can apply at once to be on that Register are set out on the leaflet. Firstly, "They have got to be at least twenty-one years of age." I have had a letter in to-day saying that twenty-one is too young. (Laughter.) That is a question I shall leave to the matrons to decide; I am not going to do it. Secondly, "They have got to be of good character." I think that stands to reason. Thirdly, "They have got to hold a certificate or certificates of three years' training in a nurse-training school or schools recognised by the Council for the purpose of admitting practising nurses to the Register of the College"; or they have got "to hold a certificate of not less than two years' training in a nurse-training school recognised by the Council for the purpose of admitting practising nurses to the Register, followed by at least two years' *bona fide* practice as a nurse." There are a certain number of nurses here who will recognise the reason for that. Or, fifthly, they have "to produce evidence of training to the satisfaction of the Council, having regard to the date at which the training was taken, followed by at least five years' *bona fide* practice as a nurse." The reason for that last clause was that there may be some among the older nurses who have not got certificates of three years' training or two years' training, but whom everybody would recognise as *bona fide* nurses who ought to be on the Register.

### The Composition of the Council.

Then, acting on the advice of Sir Charles Russell and Mr. Vesey Knox, K.C., who have both been good enough to give us their services in this matter, we have worked on the basis of the Bill which has been already promoted, as I said before, and we have tried to simplify it in every possible way. Parliament, in these more strenuous days, is much more inclined—at least so I am told—to entrust a body such as this with general powers, and not to waste the time of Parliament by arguing about the rules and regulations, but to leave them to be approved by the Privy Council. We have therefore put a great part of what was in the other Bill, a certain amount of which was contentious—we have put that under Section 4—that "Rules may be made under this Act." The representatives of the Central Committee for State Registration pointed out that the word "may" was to their mind rather doubtful, and suggested that we should put in the word "shall." That is a question of parliamentary drafting which we need not go into. But then the most important point in the constitution is this: we want this College of Nursing to hold practically the same position in the nursing profession as the Royal College of Surgeons and the Royal College of Physicians do with regard to their respective professions. We also think that, especially in this democratic age, it is right that the government of the Council should be entrusted to the nurses themselves—(applause)—and I think nobody will disagree with that. The nurses have done most splendid work during the past two years. If they are fit to do that work, and if they are fit to have a College at all, then they are fit to govern the College. (Applause.) But, of course, this is rather a different profession from some of the others, and there is a very large mixture—there must be—in administration with the lay element and with the medical element. We have therefore provided that the Council shall be composed as to one-third of members nominated by the Privy Council and Government Departments, probably the Local Government Board, of course, and the medical profession, and that the other two-thirds shall be elected by the nurses themselves. That, I think, is a right proportion; it gives a fair share of lay and medical representation, and it gives the nurses practically self-government.

Then I need not go into all the other rules; it practically covers the whole of the work that the College would be required to perform. The rules made under this Act have got to be approved by the Privy Council.

**THE COLLEGE OF NURSING: Full Report**—(*continued*).

### A Nurse's Right of Appeal.

There is one point that I think I ought to take up. It is a letter from Mr. Frankau, of St. George's Hospital. We have taken power under the rules to get a Register, and, of course, we have taken power to strike any nurse's name off that Register. Mr. Frankau suggests: "I propose to insert a clause giving any nurse whose name under a General Council Order shall be removed from the Register a right of appeal to an independent tribunal, say, of three barristers of not less than seven years' standing, their decision to be final." Well, our position, of course, will be that in case of any nurse being struck off the Register that will be a very serious thing, and will mean the loss of her livelihood, and. therefore, that there shall be a right of appeal, and that right of appeal shall be as little expensive as possible. We ought to make it possible for any nurse to appeal, and for her not to be prevented from appealing by any monetary consideration.

### The First Council under the Bill.

Then Section 5 represents a point which really is the point of agreement—at least I hope it will prove to be so—between the College of Nursing and the Central Committee for the State Registration of Trained Nurses. They felt, and, I think, very rightly—I am bound to say I entirely agree with them—that it was rather hard that they who had fought this question for twenty-five years should suddenly find another body coming along who had had very little to do with it in the past, setting them on one side and taking such credit as there may be in obtaining the State registration of nurses. I think we all agree that it would be very unfair that they should not have a voice in the first Council appointed under a Bill which their efforts have done so much to gain. We therefore agreed that on the passing of this Act the first Council should be appointed, one-third by the State in some form or other and by the medical profession, one-third by the College of Nursing, and one-third by the Central Committee for State Registration. In the Bill it is put that that Council is to hold office for one year, but at a discussion yesterday we decided that it should hold office for two years. The reason for that is that one year will be a very short time after the passing of the Act in which to get the whole machine into working order, and it would be very advisable that the same Council shall be in control for two years. Some of them wished for three years, but a reason why we did not want that was just this: in three years' time what we have called the close time comes to an end. For three years now It is within the power of the Council to put anyone's name on the Register whom they hold to be a *bona-fide* trained nurse. At the end of three years' time only those nurses' names can be added to the Register who have passed the various examinations and tests which may have been set up in the meantime by the College. They thought the first Council ought to hold office for three years, and the reason why we did not agree was that we thought it very advisable, when that three years is over—that is to say, when the College may be looked upon as being in working order —that it should then be under the elected Council, under the Council which is elected as to two-thirds by the nurses themselves; in other words, that in three years' time the whole machine should be working in the permanent way—in the way it is intended to go. That solution, I think, commended itself to them, and I think it will also commend itself to those of us who have been working on this Bill.

### A Nurse on the Register becomes a Member of the College.

Then there is the important question of fees. Section 6 provides that "Every candidate for examination or registration shall pay to the College of Nursing such fee as may be prescribed by the Rules." Those of you who have had our Memorandum and Articles of Association will have seen that under these Articles we said that people were entitled to come on the Register of the College, and that everyone on the Register of the College was entitled to become a member of the College on payment of £1. But I think we got it wrong. That would have meant two things. You would have had your State Register, and you would have had your College Register, because the people who had already got on the Register might not all wish to be members of the College. We are therefore altering that, and the position now is that every nurse who is entitled to be on the Register can come on that Register on payment of £1—either £1 down or 25s. spread over five years. We get a little bit of a pull in the interest. (Laughter.) Every *bona-fide* nurse is entitled to come on the Register, and the minute they are on the Register they become *ipso facto* and without further payment members of the College, and are therefore entitled to vote for the two-thirds of the governing Council. That is the first and last and only payment that the nurses will be required to make. In the old Bill there was a yearly payment of 2s. 6d., I think, but we have done away with that. We felt that it was rather anomalous that a nurse who was once on the Register should be removable either because she had been guilty of grossly unprofessional conduct or because she had failed to pay 2s. 6d. on January 1. I want to make that perfectly clear—that any nurse who is on the Register has only got to pay £1 down, or 25s. spread over five years, and she becomes a member of the College and is entitled to a vote for the governing body of the College. Well, I do not think really that I need go into the rest of the Bill; that is only formal. I think that really says all that I have got to say about the Bill, though I shall be very glad to answer any questions afterwards.

### How to get the Bill Passed.

The next item in our letter was the formation of the first Register of members of the College. Now we want to proceed with the formation of that Register at once. Application forms have been got ready and will be sent out, and I do ask all those nurses themselves, all those entitled to be on the Register, to come on this Register as soon as possible. The reason is this. We want to get the Bill passed as soon as possible. The larger the Register with which we go to Parliament, the easier it will be to get it. Of course, it is almost impossible to pass a Bill in these days—in fact, it is absolutely impossible—unless you can get the Government to take it up. But I am not without hope that if we can go with an agreed Bill, if we can say that this really expresses the opinion of the nursing profession, the medical profession, and the governors of hospitals, and if we can go with a large Register, with, say, 10,000 or 15,000 nurses behind it, then I am not without hope that we may be able to get a Bill through, even in these days.

### The New Offices of the College.

Miss Rundle, who is known to a good many of you, has kindly accepted the post of Secretary, under Sir Cooper Perry, who will remain Honorary Secretary, and a gentleman has been kind enough to lend us two or three rooms in Vere Street. We shall open the office there at once, and those of you who wish to forward this movement cannot possibly do better than send in your names and your sovereigns. The reason, I ought to say, for going to Vere Street is this: There is a site there, a site a good many of you may know, the site of the old Post Office, which would be in many ways an admirable site for this College. It is very central, it is near a lot of the Nursing Homes, and, if that is any recommendation, it is near the doctors in Harley Street and Wimpole Street. (Laughter.) And what perhaps is a bigger recommendation, it is near the Tube station and the 'buses, and it is opposite Marshall and Snelgrove's—which also may have its attractions. (Renewed laughter.) But without tying ourselves down—of course, we are in no way committed—I may say that it would be a very central and a very good site if we could have it. It is just as well, therefore, that we should begin in Vere Street if the College itself is eventually to be in Vere Street. So that, in view of the possibility of our eventually having this site, we have had these offices lent us in Vere Street, and we propose to commence business there at once.

### The Formation of the Consultative Board.

Then the third item in the letter of invitation is the one we are really here to-day to discuss, and that is the formation of the Consultative Board. Now, I have explained the formation of the Council, with its Government representatives and with its representatives of the nurses themselves; but we did feel very strongly that, after all, this registration of nurses, the settling of a curriculum, the arranging for examinations, and all these questions, are matters which very vitally concern the governors and managers of the hospitals, and we thought it was right, therefore, to make provision for taking their advice on those important questions of principle. We suggested that every recognised training school and Poor-Law infirmary which is a training school should be asked to send representatives, and that out of those representatives should be formed a Consultative Board. Well, we now find that if they all did send representatives they would amount in all to some four hundred or four hundred and fifty people. Well, of course, that is too big a body for a Board, but, on the other hand, it is very advisable that we should have as wide an expression of feeling as possible in order to know what is the opinion of the various training schools throughout the country—what is their real opinion as to the various proposals we put before them. We have suggested, as you will see, two alternatives. The first alternative would be that the number should remain as we had it, at four hundred and fifty people, or somewhere in that neighbourhood, who should meet possibly every three months just now, while there are important questions to settle, and who eventually would meet probably once a year at different places in the country—in fact, it would be turned into something very much like the yearly Conference of the British Medical Association; it would meet one year in London, another year somewhere up in Yorkshire perhaps, and I hope it would meet in Lancashire some time or other; another year it would meet at Cardiff—it would meet in different places, and it would turn itself really into a yearly Conference on questions connected with the nursing profession. That, to my mind, would probably be the best solution, and would probably give us what I said we particularly desired to have: the widest possible expression of opinion.

The other alternative is that out of the representatives of the training schools should be chosen a smaller committee, say, a hundred, who could meet more frequently, and whom the Council could summon whenever there was any question needing their attention. I shall put those alternatives to the meeting here to-day, and I hope ladies and gentlemen will express their opinion, or they may have even a better solution, and we shall try to-day to settle the manner in which the Consultative Board will be formed.

I want just to clear one point up. I have had one or two questions asked as to why there is a Consultative Board and a Consultative Committee. That is only quite a small question of internal administration. Obviously, for the consideration of a large body such as the Consultative Board, it is necessary to have a carefully prepared agenda drawn out, and that is the only purpose for which the Consultative Committee exists; it exists to prepare the ground and the work for the Consultative Board.

### A Central College with Branches in the Dominions.

Well, ladies and gentlemen, I really think that is all I have got to say. I am glad to tell you that the matter has already been very actively taken up in Scotland. We have taken provision for forming branches in various parts of the United Kingdom, and the matter has been actively taken up, as I say, in Scotland, where I think they are even more advanced than we are. The matter is also being taken up in Ireland, and I have reason to think that we shall be able to get a satisfactory settlement there; and I am glad to say I have had letters from an influential gentleman in Winnipeg who is anxious as soon as we get going to start a branch of the College for Canada. He thinks, and I agree with him, that if we can only get this central College established here, with branches in the various Dominions overseas, we shall be able to do almost all we want for nurses and for the nursing profession.

### Funds for the College.

Of course, there comes the rather important question of funds. Well, with your approval, if we get a satisfactory arrangement about the Consultative Board to-day, I think that it is a very proper course for us to take—to ask the public, who have done so much for the men of the Empire, and rightly done so much, whether they will not also do something for the women who have done such gallant work in this war. (Applause.) I have had happy results already. I have only approached one or two people, and they have promised to give (Hear, hear.) I saw a gentleman, yesterday, who has been instrumental in getting up a sum of £30,000 for a convoy of ambulances for the Red Cross, and he asked me whether there was anything else I wanted money for. I could not think of anything else for the Red Cross at the moment, although it is not often I cannot think of something wanted for the Red Cross—(laughter)—so I said, "Oh, well, give me something for the College of Nursing"; and he promptly gave me £500. (Applause.) And further, although I am not authorised to state this as a fact, I may say that I am not without hope that those ladies who have raised, or undertaken to raise, £100,000 to build and equip the "Star and Garter" may be willing to help us also.

### THE COLLEGE OF NURSING: Full Report—(*continued*).

A MEMORIAL TO NURSES' WORK IN THE WAR.

Well, ladies and gentlemen, we do invite you this afternoon to express an opinion. This is only the first draft of the Bill, and we are very anxious to tell you that it is not the final form, and if you can assist us in improving it in any way we shall be only too glad. I cannot help thinking that when we do go to the country they may take our view, that as at the end of the Crimean War the happiest memorial, the finest memorial, was the Nightingale School, which is housed in this building—as the whole science of modern nursing practically owes its origin to the work of Florence Nightingale during the Crimean War—so the organisation, the co-ordination of the nursing profession and of nurses' work may owe its origin to the glorious work done by the nurses in this great Eurpean War. I think if we can do this, or, rather, if the nurses can do it for themselves, it will be the finest memorial we could have, and it will have enabled us to do our share in the war, so that out of evil will come good; and we shall have something which will be for the lasting benefit not only of the nurses themselves, but also of the nation, the Empire, and the whole world. (Applause.)

### THE DISCUSSION.

Dr. BEZLY THORNE (Royal British Nurses' Association): Mr. Chairman, ladies and gentlemen,—I have practically nothing to add to what you have heard from Mr. Stanley. I am not in a position at the present moment to commit the Royal British Nurses' Association to any definite course of action, but the governing body of that Association has met and decided by a unanimous vote that it should adopt the most benevolent attitude possible towards the College of Nursing, and it is with the approval of that body that I have accepted the post of one of the Vice-Presidents of the College of Nursing. I hope that before very long conferences will take place which will lead us to a still closer union in which the two Associations may be able to go hand-in-hand with the Central Committee for the State Registration of Nurses towards the attainment of a Bill which will place the nursing profession on a sound footing of organisation, for which we have been fighting now for nearly thirty years. I may say that we have already got a Register of Nurses which comprises about 3,000 nurses; we have established an examination for a diploma which we consider to be the *cordon bleu* of the nursing profession, and, in addition to having our own nursing organisation in this country, we have already a prosperous and steadily increasing branch in Australia; and my earnest hope, speaking as an individual, is that all these forces; and all these means of working, will be brought into co-ordination with the College of Nursing. (Applause.)

The CHAIRMAN: Mr. James Blossom will move a resolution on behalf of the Poor-Law Officers.

POOR-LAW OFFICERS ASK FOR MORE REPRESENTATION.

Mr. JAMES BLOSSOM (Poor-Law Officers' Association): Mr. Chairman, ladies and gentlemen,—I do not know that I have a resolution to move, but I have certainly been asked to speak here this afternoon on behalf of the Poor-Law representatives at this meeting. I would like, Sir, in the first place, if you will permit me, to thank you for your very excellent and lucid exposition of this Bill, and to say on behalf of the Association that we are heartily in favour of this Bill, provided that Poor-Law institutions are secured adequate representation on the Consultative Board and the Council. I say that because on the list of the Council members, which has been placed in our hands, out of twenty-five members there are only four that represent Poor-Law institutions, and we do not think that an adequate representation. May I be permitted to point out, Sir, that in Poor-Law infirmaries there are 94,000 beds, while in all other hospitals, including general, special, fever, and smallpox, there are only 84,104, so that really the Poor-Law institutions are looking after the largest number of sick persons of any organisation in the country? You may at once reply that of course all those are not training schools for nurses, and that is correct. The number of beds in our infirmaries which are training schools are 50,606, and the staff is:—

| | |
|---|---|
| Matrons and Assistant Matrons ... ... | 234 |
| Sisters and Charge Nurses ... ... | 921 |
| Staff Nurses ... ... ... ... ... ... | 292 |
| Probationers (who are, of course, undergoing training) ... ... ... ... | 4,452 |
| Making a total of ... ... ... | 5,899 |

The training schools of Poor-Law institutions add to the list of nurses every year by probationers who have completed their training 1,368. Now, whilst we are in absolute sympathy with the objects and purposes of this Bill and of this College, we do feel that on the Consultative Board and on the Council these Poor-Law institutions should be adequately represented, and I am commissioned, Sir, just to say that at the meeting to-day, and that we trust that some means will be found by which this representation may be secured. (Applause.)

POOR-LAW NURSES CAN BE PROPERLY REPRESENTED.

The CHAIRMAN: Perhaps I had better take up the questions as they arise; that will be the simplest way. I should like, in answer to Mr. Blossom, to say that we are very anxious in every possible way to give proper representation to the Poor-Law nurses; but this Council that he is asking about now is only in existence till the Bill is passed. When the Bill is passed there comes in the Council I have mentioned for two years, on which the Government and the medical profession have power to appoint representatives; and after those two years the matter is entirely in the hands of the nurses themselves. If, as Mr. Blossom says—and I quite believe he is correct in what he says—if they have a larger number of nurses than others, then they can elect the whole of the Council if they like—(laughter)—the matter will be entirely in their hands. I thank Mr. Blossom for what he has said.

At a later stage Mr. TOM PERCIVAL, of the same association, declared that though at the end of two years the Council would be elected by the nurses, these first two years would see the foundations laid of this great scheme, and that at the end of two years any attempt to make alterations in the procedure would be a matter of considerable difficulty.

POINTS ON THE DRAFT REGISTRATION BILL.

Major RHODES (North Stafford Infirmary): On the question of the constitution of the Consultative Board, I

have been asked by my Committee to say that we are in favour of the smaller body being formed, and we suggest that at least seventy of the hundred should be representatives of the provinces—or rather I would say representatives other than from London—(hear, hear)—and that the meetings should be held quarterly, and that they should be half-yearly in London and the other two in one or other of the big towns out of London. As to Section 3 of the Bill. You particularly mention keeping a Register of Mental Nurses; and my committee wondered why you particularly mentioned mental nurses, and whether amongst the Register of Nurses you included fever nurses and all special nurses, such as children, eyes, orthopædic, and so on—that is, nurses having had other than general training.

With regard to Section 10, we were not quite clear about the wording of that, and I shall be glad if you will kindly explain it to us. "A prosecution for any of the offences in this Act mentioned shall not be instituted by a private person without the consent of the College of Nursing, but with such consent may be instituted by the College of Nursing." It looks as though on the wording of that only the College of Nursing can institute a prosecution. The next point I should like to mention is with regard to Section 11: "Nothing contained in this Act or in any rules made thereunder shall confer any authority to practise medicine or to undertake the treatment or cure of disease." My committee think that there should be a further clause added in that respect, in respect of registered nurses purporting to be able to practise medicine or to undertake the treatment or cure of disease, and that the punishment of such misrepresentation should be being struck off the Register. I do not think I have anything further to add.

The CHAIRMAN: I will ask Sir Charles Russell to answer as to those points.

Sir CHARLES RUSSELL: With reference to Clause 10, the object of that is to prevent frivolous or vexatious prosecutions of nurses under the Act; therefore it lays down that no prosecution shall be instituted without the permission mentioned in that clause.

A REPRESENTATIVE: I do not quite follow Sir Charles Russell. According to Section 10 no one can prosecute without the consent of the College of Nursing, but if the College gives consent, then the prosecution must be by the College, so that no private person can prosecute at all.

Sir CHARLES RUSSELL: Oh, that last line is obviously a misprint. The printer has somehow gone wrong there.

The CHAIRMAN: I will ask Sir Cooper Perry to deal with the other point, as to Section 3.

### THE POSITION OF MENTAL NURSES.

Sir COOPER PERRY: I think the answer to that, Sir, is that there is already in existence a Register of Mental Nurses, which is kept by the Medico-Psychological Association, and on that there are a large number of nurses, all of whom have gone through a long curriculum. In the draft Bill, which is only a first draft, it is put, "if they think fit." It is a matter for consideration whether those mental nurses would like to come on to the Register, and if they come on to the Register, whether they would like to be on a separate Register. That is a matter for discussion with that Association, which has already done very excellent work in that direction. As regards the question of fever and other special nurses, that also will require consideration. In the Bill which has already been before Parliament you can be registered with the addition that you have special practice or special knowledge of fever nursing, but no fever nurse is registered as such; she must go through the full curriculum, although she may have, after she has been through the full curriculum, a special training in fever nursing. But all these branches will require very careful consideration by the Consultative Board. There is the special nurse trained in children's hospitals, and there are special nurses of every kind, and the Bill makes no attempt to decide those questions in its present form, which is merely, as has been said, a first draft. When a conclusion is reached it may be possible to include all these various kinds of specially trained nurses.

### THE CLAIMS OF SPECIAL NURSES.

Dr. VOELCKER (Great Ormond Street Children's Hospital): Mr. Chairman, ladies and gentlemen,—Sir Cooper Perry has already answered one of the questions I am very anxious to have some light upon, and that is, What is to be the position of nurses who have devoted themselves exclusively to the nursing of children? There are a certain number of women who wish to devote themselves exclusively to the nursing of children, just as some devote themselves to nursing mental cases or fever cases exclusively; and it seems to me that it would be unfair if special advantages for registration were given to nurses who so devoted themselves to mental cases, and similar advantages were not given to those who devoted themselves to children. So that it seems to me it would be possible to grant a special register as in the case of the mental nurses—to grant permission to be put on a special register for children or that some arrangement might be made by which a woman did not have to work six years at least before she was entitled to become a registered nurse.

I am quite prepared for a certain amount of criticism, but I am perfectly sure of my own view on the point, that women require three years' training before they are adequately trained children's nurses. On this ground I hope that the Consultative Board will consider very favourably the position of women who devote themselves to nursing children.

The CHAIRMAN: That, of course, will be one of the questions which the Consultative Board will have to consider very carefully.

Mr. DEACON (Chairman of the Royal Infirmary, Liverpool): Mr. Chairman, ladies and gentlemen,—There are one or two points in the Bill to which I should like to draw attention. I regret that, owing to the short time which has elapsed since the Bill was in our hands, and also the circular, which is only dated the 7th instant, we have not been able to have a meeting of the Infirmary Committee to consider the matter. I happened to be away from home for a day or two, so that I did not get it until the 10th, when it was quite impossible to call a meeting, because one cannot call such a meeting at a moment's notice. I think if we are to have the consent of the governing bodies it would be well that a little more time should be allowed. (Hear, hear.)

With regard to Clause 4, Sub-section (1) (iii), I understand that the intention of the College of Nursing is to recognise the certificates of certain training schools, and I presume the recognition of the training schools will come under Clause 4, Sub-section (1) (iii). There is no particular clause, so far as I can see, which gives specific power to recognise training schools. It does not appear that there is any authority given to the Council to recognise training schools, except under that clause.

### THE COLLEGE OF NURSING: Full Report—(*continued*).

### The Training School's Position.

Then I should like to ask this, Sir: if the power to recognise training schools is to be entirely, as I understand, in the hands of the elected Council, and if that Council refuses to recognise any particular training school, what is to happen? You have already said, Sir, that nurses struck off the Register will have a right of appeal. Now, suppose the Council decides that some particular training school shall not be recognised: is that training school to have a right of appeal, and if so to whom? Take my own hospital, for instance; we have rather over 300 beds. I do not know whether it would be recognised or not, and no one has been able to tell me. I presume it will. But take another hospital with sixty, seventy, eighty, ninety, or one hundred beds. If such a hospital were not recognised, would it not feel very much aggrieved unless there were an independent tribunal which could settle whether it was to have recognition or not? Therefore it seems to me there must be some method of appeal against the decision of this elected body, over which the person censured or cut out has no possible power.

Then as to Sub-section (2) of Clause 4, it is provided that: "Rules under this Act shall be made by the Council, but shall have no effect until they have been approved by the Privy Council." I do not know what right, Sir, anybody has to appeal to the Privy Council. I do not know whether an individual, for instance, can appeal. I do not know sufficient about the law to answer the question, and I should like just to be informed whether when the rules are submitted to the Privy Council, and before they are approved by that Council, a hospital committee or body of nurses or anybody else has a right to lay before the Privy Council such arguments as they see fit.

With regard to the general question, Sir, of the size of the body for a Consultative Board, I personally think —it is a mere personal opinion—that the larger body is wiser under the circumstances. A consultative body of 100 is of no use as a consultative body. It simply means we want to meet and discuss things generally, as I understand, and I therefore think the 450—by means of which all the hospitals in the country would be represented—would be wiser than some little body of 100 or so. I think that perhaps when the thing is once running smaller meetings would probably answer all the purposes.

### Right of Appeal for the Training Schools.

The Chairman: May I just say a word in answer to some of the points Mr. Deacon raised? With regard to the recognition of the training schools, I quite agree that there ought to be some right of appeal, and that we shall have to provide for that. As regards private people or any hospital governing body having a right of appeal to the Privy Council on the question of the rules—or, rather, to go to the Privy Council before rules are made—Sir Charles Russell informs me that they would have that right. As regards the shortness of notice given, Mr. Deacon, it appears, was away from home. Well, that was not our fault. We certainly did not give a very long notice, but it is very essential that we should get ahead with this work as soon as possible. After all, it has been waiting twenty-five years. And there is this much to be said: that if we do not get this properly organised and started before the end of the war I do not think we shall ever do it at all. There will be a large body of people coming back after the war, a large body of nurses, who will require help of various kinds. If they come back and find a properly organised Association it will be of the utmost value to them. There is also the other question that there will be a large body of partly trained people who will come back, some of whom may compete with the nurses. In my opinion it is most essential that we should get ahead with this matter as soon as possible, but I promise that in future we will give as long notice as we can.

### The Election of the Consultative Board.

There is another point—namely, the method by which the Consultative Board is to be elected. It seems to me that by adopting either of the proposals on the agenda we fall between two stools. If you are aiming at a large body, then the number suggested is not large enough, because I think there should be opportunity given to every member to attend the Annual Conference. If, on the other hand, you are aiming at a smaller body, then first of all it seems to me 100 is too large a number, and in the second place it is difficult to make a selection from a large meeting like this; indeed, I think the difficulty is so great as to be almost insuperable. We do not know one another, and if we have 100 or 200 names put before us how do we know who are the best people to vote for? I venture to suggest very respectfully that you should carry forward still further the plan you have adopted of following the procedure of the British Medical Association. You take the British Medical Association as the model for your title, and for several details of the scheme. Why not adopt their principle of dividing the country into districts? Let the districts meet and settle local difficulties, subject, of course, to the ruling of the central body, and, further, let these districts send up representatives to the Consultative Board, which will, of course, deal with the country as a whole. I suggest that by that means you will secure interest in the scheme right throughout the country; you will secure the interest and the attendance of all who are interested in these questions—medical officers, matrons, and nurses; while by giving the districts power to send representatives to the central body you will secure on that body adequate representation for the entire nursing community. (Applause.)

Mr. W. Domville: Mr. Chairman, ladies and gentlemen, I just want to say two sentences. First of all I want to express my sympathy with the remarks of the Chairman of the Liverpool Royal Infirmary, and his desire to secure proper recognition of the teaching and nurse-training schools. I have been for forty years connected with a large nurse-training school in Exeter, and we are very jealous of our privileges. I should be glad to know the conditions under which training schools are to be recognised by the College of Nursing, and whether we are to understand that an invitation to this meeting constitutes a recognition on the part of the Council that we are recognised as training schools.

### Representation of the Medical Profession.

Then also I should like to say this: A good deal has been said this afternoon about the British Medical Association, but I notice that this is entirely dropped out of the Bill, and that there is only a vague reference to the medical profession. I venture to say it would be wise if in Clause 5 it was provided that three members should be directly nominated by the British Medical Association, as recognised in previous Bills which have been before the House on more than one occasion, because this would save a lot of uncertainty in regard to the very uncertain expression about one-third being appointed by the Privy Council or the medical profession. I suggest, therefore, that it would save a good deal of misapprehension if, in that clause, the nomination of three members out of the number to be appointed by the Privy Council or other bodies were directly given to the British Medical Association, who, together with the Central Committee for the State Registration of Nurses, have worked for the last twenty-five years to secure the objects for which we are met here to-day, and for which this College of Nursing has been founded.

The Chairman: The first point raised by Mr. Domville—namely, the recognition of training schools—is, of course, one which the Council will have to go into most carefully. That is one of the objects with which the College has been founded. I am afraid I could not possibly express an opinion upon it now.

As regards the British Medical Association, we did not put it into this first draft of the Bill. The medical profession has obviously got to be represented, and we shall have to discuss with them the way in which they wish to be represented, and it is probable, of course, that it will end by its members being nominated by the British Medical Association.

I need not now take up Mr. Percival's point about the division into districts, because I shall have to put that to the meeting afterwards.

### The Smaller Provincial Hospitals.

Mr. Batchelor (Wharncliffe Military Hospital, Leamington): Ladies and gentlemen, I am here as representing one of the smaller provincial hospitals, and I should like to endorse the remarks Mr. Wade Deacon made as to the importance of some court of appeal in settling the standard of each hospital as to their competency to grant certificates for nursing. The hospital which I represent is, no doubt, a type of many others. Although a small one, it is thoroughly up to date in staff and equipment; and I would remind you, though it is scarcely necessary, I feel sure, that in some of the smaller hospitals more individual attention can be given to the training of nurses, perhaps, than in the larger institutions. If these smaller hospitals are excluded from competency, or from the list of those competent to give certificates, the question will arise, how are they to get any probationers to come to their hospitals? Of course, it would be obviously impossible.

The Chairman: There is no intention of excluding such hospitals.

Mr. Batchelor: I bow to your decision, Sir, but I may say there is a very general feeling that the smaller hospitals will be excluded from granting certificates. I am very glad to hear that that is not so.

The Chairman: That question has to be gone into by the Consultative Board, but I do not take it that they are to be excluded. There is a lady who wishes to ask a question, but, before she does so, may I say that what we have to decide this afternoon is the formation of the Consultative Board? All these other questions are very interesting, and will have to be threshed out, but we cannot get on with our machinery until we have settled what form the Consultative Board is to take. Now I shall be glad if the lady will put her question.

Miss Benjafield: I just want to be clear about the question of the examination for nurses. Is the College of Nursing going to institute an examination, and are the examinations now held at various training schools to be abolished and the same certificate given by the College of Nursing to all candidates, whether trained at hospitals or at an infirmary? I should like to know that, Sir.

The Chairman: I cannot answer that. I do not know. (Laughter.) It is one of the things the College has been founded to try and settle. It will be settled, but as to how it will be settled I have not the faintest notion.

### Various Questions.

Now, are there any other speeches? I have had three questions passed up by a lady who is anxious to save the time of the meeting, which is very kind of her. The first question is: "Will all nurses who pass the same examination get the same certificate?" I should say, decidedly. I will risk that. (Laughter.) The next question is: "Does 'all branches of women's work connected with hospitals,' except doctors and midwives, include dispensers, clerks, and domestic staffs, such as cooks, sewing-maids, wardmaids, and scrubbers?" I am not quite certain about the scrubbers—(laughter)—but if this College gets to be what it ought to be it will give certificates for every branch of women's work connected with hospitals. Nothing could possibly be more important than a certificate to the cook. ("Hear, hear," and laughter.) "Will two-thirds of the Council always consist of matrons or trained nurses, or only two-thirds of the first nominated Council? Will the registered nurses be able to elect to it any persons they choose?" Yes, anybody they choose. It will be a postal ballot, a private ballot, and it will afford a splendid chance for the nurses to vote their matron off it if they do not like her. (Laughter.) "If from 25,000 to 100,000 registered nurses are to elect future Councils, will not the canvass of such large numbers, either by circulars sent by post, by addressing meetings all over the country, or by calling on matrons and influencing their staffs through them, be so great a tax on both the time and the money of the best and most occupied matrons as to give an unfair advantage to candidates who are at leisure or who happen to have control of funds?" Well, that is the same thing that happens in regard to elections everywhere. I have heard that some of the richest and most influential men have even managed to get elected members of Parliament. (Laughter.) But I do not think we need take much account of that. Every nurse will have a vote, and I do not think you could get a much wider electorate than that. I do not see the matrons going about canvassing for votes, and I do not think they are likely to.

### The Consultative Board: Three Alternatives.

Now the question really before this meeting is this one about the Consultative Board. The Council itself actually is to form the Consultative Board, but we only want to get an indication of how the representatives of the training schools wish that board to be formed. There are three alternatives as far as I can see now. One is to have a big meeting—450 or 500, I do not know how many it may be—say, two representatives of every recognised training school—to meet quarterly now, while so many questions have to be settled, though eventually probably once a year will be enough. Another way is to have a smaller body, say, 100, chosen from those representatives by machinery which we should have to settle. The third way is Mr. Percival's—that is to say, either by counties or districts. And I think, just in order to try and get an expression of opinion, I will put the last one first. Will those who think it would be a good thing to arrange for the Consultative Board to be elected by districts or counties hold up their hands? (A large number of hands were held up for this proposal.)

A Representative: Will you put it for or against?

**THE COLLEGE OF NURSING: Full Report**—(*continued*).

The Larger Consultative Board Agreed to.

The Chairman : Well, it is difficult to count; we only want to get a general impression. Still, I will put it. Those against election by districts please hold up their hands. (A much smaller number of hands were shown.) Now how about the smaller number, say 100, to be chosen, to meet at fairly frequent intervals—probably at first about once a month? Those in favour? Nobody seems to care for that idea. Now, then, as to the bigger body—that is to say, comprising representatives of all the recognised training schools, meeting probably quarterly, or whenever required, for the next few months, and afterwards twice a year, or once a year, in different places, beginning with London. Those in favour of that? Well, there is no doubt that the last, the larger body, is the most favourably received.

A Representative : With representatives from each training school?

The Chairman : Yes.

Mr. Langley (Stoke) : Might I suggest that either in proportion to the number of beds or the number of nurses in training the representation should be on this Consultative Board—say, one for every 100 beds? It is hardly fair that a hospital with, say, sixty beds should have the same representation as St. Thomas's.

The Chairman : Well, I am afraid I do not think I can, at all events now, agree to that. After all, the questions that came up might concern the hospital with sixty beds very much more than St. Thomas's. It is not so much a question of voting; what we want at this meeting, with regard to the Consultative Board, is to get an expression of opinion which we can act upon. I do not think that would be possible. Is there any other member who wishes to speak?

Male Nurses.

A Representative : Is the question of male nurses to be considered at all?

The Chairman : Yes, it is. In the Bill before Parliament now the male nurse is put separately. We left it out in this Bill, because we did not think it necessary to treat the question separately—that is to say, "nurses" includes everybody. But I have heard arguments since which have caused me to think it might be necessary to deal with male nurses separately.

The Representative : I am very strongly of opinion that that would be absolutely necessary.

Mr. Leech : May I ask whether it is the intention of the Council that until the Consultative Board have given the Council the benefit of their views, no proceedings will be taken with regard to the Bill, or is it the intention of the College of Nursing that the Bill should be proceeded with at once?

The Chairman : We intend to proceed with the Bill at once, simply because that gives us power. As to curriculum, examination, and all these questions, we shall take no steps until we have met the Consultative Board and got their opinion. The Bill, after all, only sets up the machinery which is necessary.

Mr. Leech : May I ask this—Whether the Council will not consent to receive any immediate representation from any representative body of Governors, or administration, where such body of Governors consider that an improvement may be made in the Bill?

The Chairman : Not only from a body of Governors, but we want it from any individual. This is only the first draft which we have got out, in order to give you an idea of the lines on which we are proceeding.

Should Nurses be Tested by Examination or Record of Work.

Mr. Charles Lupton (Treasurer of the Leeds General Infirmary and Lord Mayor of Leeds) : May I ask if it is the intention that all nurses of schools who fall in with this scheme should necessarily have their own diploma by examination? I represent a very large training school in the North of England, and we are strongly of opinion that the best qualities of a nurse are most difficult to test by any written examination—(hear, hear)—and from what I have heard to-day I feel that there is some danger, and also from what I have read in the papers regarding the scheme I fear there is some danger, that nurses will be tested chiefly by a written examination instead of by the record of their work. And I may point out here that the busier the school, the harder worked the hospital, from which the nurse comes, the less chance she has of perfecting herself with book knowledge, and therefore she probably has the less possibility of coming out well in a written examination, although, when you compare the one nurse with the other, the nurse who is trained at a large and busy hospital probably has much more practical experience than the nurse who comes from the smaller hospital. I was asked to mention this point by those whom I represent.

The Chairman : In answer to this question of the Lord Mayor of Leeds, I may say that all that has to be taken into consideration by this Consultative Board. Quite undoubtedly the record of training would count enormously, and the certificates gained by the nurses during her course of training would, of course, weigh very largely when the time came for granting her her diploma or certificate. These questions will all have to be very carefully considered. I quite agree with the Lord Mayor of Leeds that the written examination is not always the best test by any means.

What Constitutes a Training School.

Mr. Leech : May I take it that the Council of the College of Nursing will settle what are to be considered training schools in sending out notices for the meetings of representatives of the Consultative Board? Is there likely to be any difference between the Council and anyone outside the Council as to what constitutes a training school on the Consultative Board, and if there is a probability of that, who is to settle the difference? (Laughter.) The Council, I take it. I should be quite prepared to trust the Council.

The Chairman : But another gentleman, Mr. Deacon, was not prepared to trust the Council, and therefore I accepted his suggestion that there should be some form of appeal, and I think that would be right.

In reply to a further question from Mr. Leech as to representation on the Consultative Board, the Chairman said : I am afraid I cannot look after the representation of other bodies. I think there is very little likelihood of any real difference of opinion as to what ought to be a recognised training school, and what ought not. If the Council have any difficulty they could, no doubt, ask the Consultative Committee what they thought. I cannot say that an invitation being sent out to a training school, such invitations being, of course, sent out to the best of our ability, necessarily means that that training school will be a recognised one, but I think it is highly probable that, having got our invitation now, and therefore being on the list, they will get them in the future, and that they are on the list for good and all. But, of course, it does not follow.

A question has now been handed in to me: "What about the village nurses?" That is a very difficult question, and one that will tax, I think, all the mental efforts of the Consultative Board of the Council.

Now I am asked by the Provost to announce that tea is provided in the Nightingale Home, and that everybody is cordially invited. Before leaving the hospital, I wish to very heartily thank Mr. Wainwright and the governors of the hospital, as well as Miss Lloyd-Still, the matron, for again giving us the hospitality of this splendid room. As I said before, I think it is a good omen that these big meetings should be held, in connection with this movement, in the place which is sanctified by the fact that it is the home of the Florence Nightingale School. (Applause.)

A hearty vote of thanks to the Chairman for presiding having been moved, seconded, and carried by acclamation, the proceedings terminated.

